# Development and Validation of a RP-Ultra performance liquid chromatographic Method for Quantification of Topotecan Hydrochloride in Bulk and Injection Dosage Form

**DOI:** 10.4103/0250-474X.73925

**Published:** 2010

**Authors:** P. K. Saini, C. L. Jain, R. M. Singh, S. C. Mathur, G. N. Singh

**Affiliations:** Research and Development Division, Indian Pharmacopoeia Commission, Sector-23, Rajnagar Ghaziabad-201 001, India; 1 Department of Chemistry, M. M. H. College, Ghaziabad-201 001, India

**Keywords:** Method development and validation, Topotecan hydrochloride, UPLC

## Abstract

A simple, very fast, precise and accurate reverse phase ultra performance liquid chromatographic method was developed for the determination and validation of topotecan hydrochloride in bulk and injection dosage form. A Waters BEH C18, 50×2.1 mm, 1.7 μm particle size column in gradient mode was used with mobile phase comprising of 0.1% v/v orthophosphoric acid in water and acetonitrile. The analytical column was thermostated at 50° and flow rate was set at 0.4 ml per min, with photo diode array detection at 260 nm. The retention time of topotecan was found 1.38 min. The method was validated in terms of linearity, accuracy, precision and specificity. The calibration curve was found linear between 20 to 60 μg/ml. The limit of detection and limit of quantification were found 0.2353 and 0.7131 μg/ml, respectively. Percentage recoveries were obtained in the range of 98.91% and 99.17%. The proposed method is precise, accurate, selective and reproducible. The ultra performance liquid chromatographic assay procedure, which proved superior because of its greater sensitivity and relatively shorter (4 min) run time, should be an important tool for speedy future analysis of topotecan hydrochloride in bulk and its injection dosage form.

Topotecan hydrochloride is chemically known as (4S)-10-[(dimethyl-amino)methyl]-4-ethyl-4,9dihydroxy-1*H*-pyrano[3,4:6,7] indolozino[1,2-*b*] quinoline-3,14(4*H*,12*H*)dione hydrochloride[1]. Topotecan, a water-soluble analogue of camptothecin, is approved for second line therapy of patients with ovarian carcinoma and small-cell lung cancer[2–4]. Topotecan hydrochloride is used as a treatment for a variety of human tumor-type cancers, including colorectal cancer, ovarian cancer, non-small cell lung cancer and non-lymphocytic haematological malignancies[5,6]. Topotecan undergoes both renal and hepatic elimination, with as much as 70% of dose administered gets excreted unchanged in the urine; however, more recently it was reported that approximately 40% of a dose given i.v. and 20% of an oral dose are appeared in the urine unchanged[7].

Topotecan hydrochloride and its injection is official only in Indian Pharmacopoeia[1]. Literature survey revealed that a few analytical methods were reported for the determination of topotecan using HPLC[8,9], HPLC with fluorescence detector[10] and ELISA[11]. No direct ultra performance liquid chromatographic (UPLC) method has been reported so far. In the present investigation attempts have been made to develop a very fast, accurate and precise method for the analysis of topotecan hydrochloride in bulk and from injection dosage form.

Pharmaceutical grade topotecan hydrochloride working standard (Lot No. 7NW001, Purity 96.04%) and topotecan injection concentrate 2.5 mg/2.5 ml with brand name Topotel (Batch No. 7BG004) were provided by Dabur Pharma Ltd., Sahibabad, India. Acetonitrile of HPLC grade was purchased from E. Merck, Mumbai. Other chemicals and reagents used were of analytical grade. The water used was of Milli-Q grade purified by a Milli-Q UV purification system (Millipore, Bedford, MA, USA). The instrument used for the study was a Waters, Acquity UPLC (Waters, Milford, MA, USA) equipped with a binary solvent manager, sample manager, inline degasser, and 2996 photo diode array (PDA) detector. Empower-2 Software was used for instrument control along with data acquisition and data processing.

Chromatographic separations were performed with gradient elution. All the determinations were performed at 50° using Waters BEH (Bridge Ethylene Hybrid) C_18,_ column consisting 50 mm length and 2.1 mm inner diameter with 1.7 μm particle size. The injection volume was set to 2 μl. Two mobile phase components were as follows: *mobile phase* (A) comprising of 0.1% v/v orthophosphoric acid in water and *mobile phase* (B) comprising of acetonitrile. A linear gradient was programmed as described in Table 1. The detection wavelength of 260 nm represents the wavelength of sufficient absorbance of the drug. Mobile phase was pumped at a flow rate of 0.4ml/ min with a gradient program as give in Table 1. The retention time of the principal peak was found 1.38 min. Topotecan hydrochloride is potentially cytotoxic. Great care has been taken in handling the powder and preparation of solutions. The drug is light and heat sensitive, hence the assay was carried out in subdued light and the samples and standard kept at temperature between 2-8°.

**TABLE 1:** GRADIENT PROGRAM FOR UPLC

Time (in min)	Mobile phase A (% v/v)	Mobile phase B (% v/v)
0	90	10
0.5	90	10
1.0	80	20
2.0	80	20
3.0	90	10
4.0	90	10

About 10 mg of topotecan hydrochloride working standard was weighed accurately into a 10 ml volumetric flask, dissolved and diluted to volume with diluent (equal volume of water and acetonitrile) to obtain a solution of 1000 μg/ml. Further 2.0 ml of this solution was diluted to 50 ml with diluent to obtain a concentration of 40 μg/ml. An accurate volume 0.4 ml of topotecan injection (2.5 mg /2.5 ml of topotecan) was taken and transferred to a 10 ml volumetric flask, dissolved and diluted to volume with the diluent to obtain a concentration of 40 μg/ml. The solutions used for analysis were filtered through 0.2 μm filter.

Two microlitres of standard solution was injected on UPLC system. The system suitability was checked by injecting replicate injections and found results within the range. The relative standard deviation on five replicate injections was obtained 0.5%, tailing factor 1.03, and the column efficiency 13220 theoretical plates. Two microlitres of standard and sample solutions were separately injected. The assay of the bulk as topotecan hydrochloride and injection as topotecan was calculated and found 101.4% and 103.5% of the labeled claim, respectively. The developed method was validated for the assay of topotecan as per ICH guidelines[12].

Specificity and selectivity were studied for the examination of the presence of interfering endogenous components. Topotecan standard solution of 40 μg/ml was injected. The result indicated that the retention time of topotecan was about 1.38 and none of the impurities were interfering in its assay (Fig. 1).

**Fig. 1:**
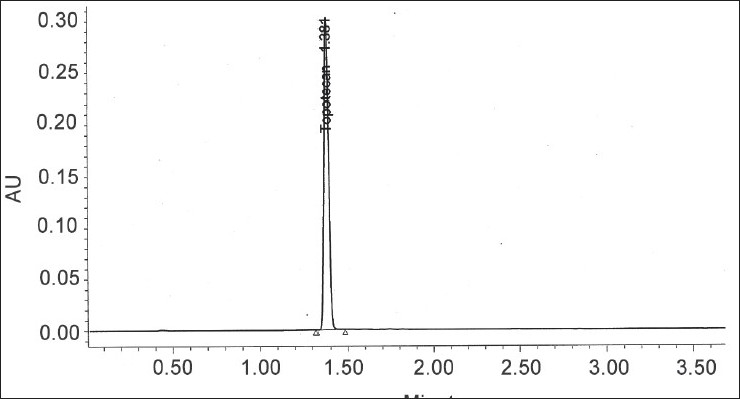
A typical chromatogram of topotecan standard

Linearity was studied by preparing standard solutions of topotecan hydrochloride at different concentration levels. The linearity ranges were found in the range of 20-60 μg/ml. The standard calibration curve was generated using regression analysis with Microsoft excel. The assay was judged to be linear as the correlation coefficient was greater than 0.995 by the least-square method.

Recovery studies of the drug was carried out for the accuracy parameter. These studies were carried out at three different concentration levels i.e. multiple level recovery studies. A known amount of topotecan standard was added into pre-analysed injection and subjected to the proposed UPLC method. Percentage recovery was found to be within the limits as listed in Table 2.

**TABLE 2:** RECOVERY STUDIES OF TOPOTECAN IN INJECTION

Label Claim (mg/ml)	Amount added (%)	Total amount added (mg)	Amount recovered (mg)	% Recovery*
Topotecan 1.0	80	0.8	1.785	99.17
	100	1.0	1.981	99.05
	120	1.2	2.176	98.91

* Average of three determinations

Precision was studied to find out intra and inter day variations in the test methods of topotecan in the concentration range of 20-60 μg/ml for three times on the same day and inter day. Precision was determined by analysing corresponding standard daily for a period of three days. The inter-day and intra-day precision obtained was % RSD (< 2) indicates that the proposed method is quite precise and reproducible.

The limit of detection (LOD) and limit of quantitation (LOQ) was calculated based on the standard deviation (SD) of the response and the slope (S) of the calibration curve at levels approximating the LOD and LOQ, LOD= 3.3 (SD/S) and LOQ= 10 (SD/S) is shown in Table 3. Robustness was determined by making small changes such as ±2% change in the volume of organic solvents of mobile phase in the chromatographic conditions, which were found not to affect the ourtcome of the assay.

**TABLE 3:** VALIDATION PARAMETER OF ULTRA PERFORMANCE LIQUID CHROMATOGRAPHIC METHOD FOR TOPOTECAN

Validation parameter	Topotecan
Range (μg/ml)	20-60
Regression equation	y=14153x−1953
Correlation Coeffi cient (r^2^)	1.0
LOD (μg/ml)	0.2353
LOQ (μg/ml)	0.7131

The proposed method is rapid, accurate and sensitive. It requires only small amounts of solvents and changing a set of conditions requires only a short time. Many samples can be simultaneously and suitably employed for the routine quality control analysis of topotecan hydrochloride in bulk and its injection dosage form. It does not suffer from any interference due to common excipients present in the pharmaceutical preparation and can be conveniently adopted for quality control analysis. We have developed a sensitive UPLC method for the determination of topotecan using small volumes of sample (2 μl). The developed method proved superior and economic because of its greater sensitivity and relatively shorter (4 min) run time. It is an important tool for speedy future analysis of topotecan hydrochloride in bulk and its injection dosage form.
